# DISPARITIES IN TYPE 2 DIABETES ONSET AMONG MIDLIFE AND OLDER ADULTS IN THE US: A FOCUS ON NATIVITY AND RACE/ETHNICITY

**DOI:** 10.1093/geroni/igae098.3449

**Published:** 2024-12-31

**Authors:** Charlotte Asiedu, Adam Davey

**Affiliations:** University of Delaware, Newark, Delaware, United States; University of Delaware, Newark, Delaware, United States

## Abstract

Type 2 Diabetes Mellitus (T2DM) disproportionately affects midlife and older adults in the US and globally and is marked by significant disparities across certain nativity groups (foreign-born, native-born, and second-generation immigrants (SGI)). While previous research has predominantly focused on diabetes prevalence across racial groups, there exists a significant gap in understanding how this disease prevalence and onset may differ based on immigration status and nativity. This study utilized data from a nationally representative sample of the California Health Interview Survey (CHIS, 2003–2022) to understand disparities in the onset of diabetes among midlife (45–64) and older adults (65+) in the US. Survey-weighted analyses suggested an association between nativity and age of diabetes onset (early, mid-life, and late). The study sample, N = 200,730, comprised 42.35% males and 57.65% females within ages 40-85+. The overall survey-weighted chi-square tests revealed a statistically significant association between nativity and diabetes onset (p < 0.001). Foreign-born adults had the highest prevalence of diabetes (15.1%), followed by SGI (13.3%). Foreign-born adults were also more likely to have received their diabetes diagnosis before age 40 (23.1%, P< 0.001) followed by SGI (20.9%, p< 0.001). Native-born adults were more likely to receive their diagnosis after age 60 (30.5%) compared to SGI (29.7%) and foreign-born adults (21.8%). Findings highlight disparities in T2DM prevalence and onset by nativity. The study underscores the necessity for further research on the socioeconomic, biological, psychological, cultural, and behavioral determinants of these disparities to inform targeted policies and interventions.

